# Point-of-Care Verification of Blood Culture Volume in Neonates: A Feasibility Trial

**DOI:** 10.34763/jmotherandchild.20232701.d-22-00063

**Published:** 2023-08-10

**Authors:** Justyna Romańska, Tomasz Wawrzoniak, Dominika Hołowaty, Natalia Mazanowska, Paweł Krajewski

**Affiliations:** Department of Obstetrics and Gynaecology, Division of Neonatology and Neonatal Intensive Care, Medical University of Warsaw, Warsaw, Poland

**Keywords:** blood culture, volume, neonate, monitoring and feedback, sepsis

## Abstract

**Background:**

Blood cultures remain the gold standard for the diagnosis of sepsis. However, volumes of blood submitted for cultures often do not match the recommended values. We propose a simple intervention aimed to verify the volume of blood sampled using a scale. This study was undertaken in preparation for a future, multicenter, pre- and post-intervention trial. Our primary objective was to test the feasibility (uptake and retention) of this future intervention.

**Materials and methods:**

This study was conducted at a neonatal department in Warsaw, Poland, over a period of eight months (May to December 2020). Before starting the study, we undertook an educational intervention focused on obtaining adequate blood volumes for culture. The culture bottles that were weighed in advance were distributed in all blood collection areas. Blood volume was verified by weighing the bottle immediately after blood inoculation. The calculated value was communicated to the collecting clinician and recorded. The primary outcome measure was the percentage of blood culture submissions for which the blood volume inoculated into the bottles was determined by weighing.

**Results:**

During the study period, 244 blood samples were collected for culture, out of which 205 samples were weighed (84.0%, CI_95_ [78.8% to 88.4%]). This high proportion remained stable throughout the study period. We have not observed any adverse events related to the study.

**Conclusions:**

The point-of-care verification of blood culture volume using a scale was feasible to implement. Since we have met our pre-established criterion for success, a future, definitive trial is likely to proceed.

## Introduction

The management of neonates with suspected or proven bacterial infection is one of the most common clinical problems that neonatal providers deal with in their everyday practice [1]. A blood culture remains the gold standard for the diagnosis of sepsis [1]. The blood volume cultured is directly correlated with its positivity [2]. At present, the minimum recommended volume of blood submitted for culture in neonates is 1 ml [1]. This volume results in almost 100% sensitivity, even in cases of low level bacteremia, while inoculating 0.5 ml of blood decreases the sensitivity of the test by as much as 40% [3,4].

It has been proven by numerous studies that the volume of blood obtained for culture in neonates is often inadequate and below the recommended volume [5,6]. Connell et al. found that nearly one half of blood cultures drawn from infants and children were inadequate submissions, and therefore were unable to exclude bacteremia reliably [6].

In recent decades, many quality improvement projects have been undertaken to define interventions effective in improving the process of blood culture submission [2,6,7,8,9]. Continuous monitoring of blood volumes, followed by feedback to blood collectors, has been considered to play a significant role in projects that achieved the greatest improvement [2,7,10].

Determining the pre- and post-inoculation weights of blood culture bottles is the only method applicable to the unique newborn population [6,11]. To the best of our knowledge, this method, when used in existing trials, has consisted of measurements performed by the laboratory rather than at the bedside, with periodic rather than instant feedback on the results to blood collectors. We speculate that this method, if used as a bedside tool, could be highly motivating in collecting an adequate amount of blood for culture and could provide excellent information on the diagnostic accuracy of the test in every clinical situation. Additionally, when the calculated blood volume does not match the recommended value, an attempt can be made to repeat sampling. On the other hand, the increased workload of the front-line staff that this method creates may prevent its adoption into everyday practice. We performed this study to address the question of whether point-of-care verification of blood culture volume could be incorporated into routine clinical practice in a sustainable manner.

## Materials and methods

When reporting the trial results, we followed the guidelines included in the Consolidated Standards of Reporting Trials 2010 statement: extension to randomised pilot and feasibility trials [12].

### Objectives

The primary objective of the study was to assess the feasibility of conducting a definitive trial in terms of adoption of the intervention by neonatal care providers.

The secondary objectives of the trial were as follows:

- To compare the volume of blood drawn for culture, which was declared by a collecting clinician based on visual inspection, with the volume of blood that was measured.
- To measure the volume of blood collected for culture in routine clinical practice.
- To determine the rate of true-positive and false-positive blood cultures.
- To examine, qualitatively, the acceptability of the intervention to the neonatal care providers involved in collecting blood cultures.

All of the objectives above, except the last one, were answered using quantitative methods.

### Trial design

This trial was designed to prospectively assess the feasibility of the intervention aimed to optimize the blood volume inoculated into blood culture bottles. The future, definitive study was planned as a continuous quality improvement project.

### Eligibility criteria

We decided to apply the intervention to all blood culture collection events, including: 1) obtaining umbilical cord blood samples from isolated cord segments, 2) drawing blood through umbilical catheters shortly after their placement for other clinical indications, and 3) drawing blood from peripheral vessels (through venipuncture, arterial puncture, and newly placed intravascular catheter). Blood cultures collected either from term or preterm newborns were considered eligible for the trial.

### Study setting

The study was conducted at the neonatal department at a tertiary care clinical hospital for women (Division of Neonatology and Neonatal Intensive Care, First Department of Obstetrics and Gynaecology, Medical University of Warsaw). This department sees approximately 2,000 live births annually, including 50 very low birthweight infants. There are no phlebotomists dedicated to obtaining blood specimens in this department. Instead, both nurses and physicians are responsible for collecting blood samples.

### Intervention

The study period of this trial was May 1, 2020 through December 31, 2020. Before starting the trial, educational sessions took place during clinical rounds to introduce the study to all staff. The paramount role of blood culture in the evaluation of neonates with possible sepsis was emphasized, along with the importance of collecting an adequate blood culture volume, which was defined as 1 ml at minimum. The bedside sample volume control using a precision scale was introduced. To facilitate the intervention, blood culture bottles (BD BACTEC Peds Plus/F, Becton, Dickinson and Company, Sparks, MD 21152 USA) were pre-weighed by research staff with the result of the measurement written on the bottle. We decided to use only glass bottles, since we proved that, compared to plastic bottles, the loss of weight in the glass bottles that occurred over time and under normal storage conditions was negligible. Immediately following the injection of blood into the bottle, the person who collected the sample was asked to estimate the obtained volume, and the bottle was re-weighed by another staff member (nurse or doctor) at the bedside of the patient. All the measurements (pre- and post-inoculation) were performed using a single precision scale with a read-out of 0.01 g. The weight of the blood injected into the bottle was calculated by subtracting the pre-inoculation weight of the bottle from the post-inoculation weight, with an adjustment being made for the the cap, which weighed 0.39 g. The blood volume was then determined by dividing the weight of the blood by a factor of 1.055, which represents the relative density of the blood [13]. Then, the calculated volume was communicated to the collecting clinician. Every time the blood volume sampled did not reach the targeted value, reminder educational sessions were conducted, and, if clinically justified, additional attempts at blood sampling were made. A new blood culture bottle was used if one decided to repeat sampling. The information on the volume of blood sampled for culture was available for the physician taking care of the patient to guide clinical decisions regarding antibiotic treatment.

### Outcome measures

The primary outcome measure was the percentage of blood culture submissions for which the blood volume inoculated into the bottles was determined by weighing. For this outcome, we established a prespecified criterion of 70%, which would indicate the intervention as being feasible.

Secondary outcome measures included:

- The percentage of blood culture submissions containing <1 ml of blood that would have been deemed adequate submissions (i.e., ≥1 ml), based only on the subjective assessment of collecting clinicians.
- The percentage of blood culture submissions containing ≥1 ml of blood that would have been deemed inadequate submissions (i.e., <1 ml), based only on the subjective assessment of collecting clinicians.
- The volume of blood submitted for culture among neonates in each of the following birthweight categories: ≤1500 g, 1501 g – 2500 g, >2500 g.
- The volume of blood submitted for culture in relation to the method the blood samples were collected.
- The rate of true-positive and false-positive blood cultures. A blood culture that yielded a recognized bacterial or fungal pathogen was categorized as a true-positive. A blood culture that yielded an organism included on the Centers for Disease Control and Prevention's National Healthcare Safety Network common commensals list was categorized as false-positive unless the patient was treated with antibiotics >3 days or if the same organism was identified by a culture from two or more blood specimens.
- The percentage of blood culture collectors who perceived the verification of blood culture volume as a justified action.

### Sample size

A formal sample size calculation has not been performed since it is not required for feasibility trials. We decided to investigate the feasibility of the intervention over an eight-month period to also address the sustainability of its adoption by clinical staff. In our department, blood culture collection was a procedure performed daily; thus, we anticipated about 200 events to occur during the study period. This value is larger than median study sample sizes observed in pilot and feasibility trials [14].

### Statistical methods

Statistical analysis was carried out using R: A language and environment for statistical computing, version 3.5.1., R Foundation for Statistical Computing, Vienna, Austria. Nominal variables are presented as n (% frequency), while continuous variables are presented as mean ± SD or median (Q1; Q3), depending on distribution. Distribution normality was assessed using the Shapiro-Wilk test and based on a visual assessment of histograms, as well as skewness and kurtosis values. The correlation between nominal variables was analysed using a Fisher exact test or chi-square test, as appropriate. Binomial exact 95% confidence intervals for proportions were calculated where relevant. Level of blood volume measurement between subgroups was analysed using the ANOVA and Tukey post-hoc test. All tests were based on α = 0.05.

### Ethics statement

The Bioethics Committee of the Medical University of Warsaw granted a formal waiver of ethical approval for the intervention tested in the presented study. As per routine practice in our unit, all blood samples were collected for culture only after obtaining written consent from the parent. As this quality improvement project has not been considered human subject research, informed consent was not obtained. The survey was voluntary and anonymous. The clinicians participated in the survey only after providing their verbal consent, and no personal health information was accessed outside the study center. All the data were saved as deidentified and managed after deidentification.

## Results

**Figure 1.**
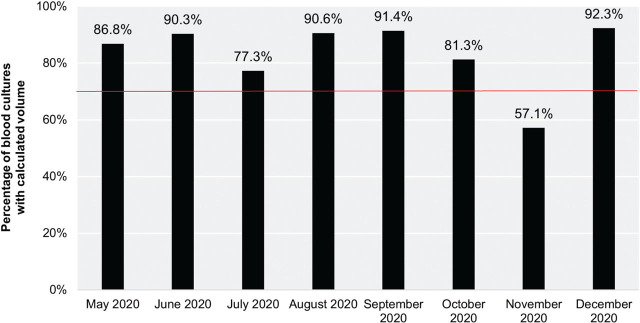
Percentage of blood culture submissions with calculated volume by month. The horizontal line represents the pre-established criterion of 70% indicating the feasibility of the intervention.

During the study period, 244 blood samples were collected for culture, out of which 205 were weighed (84.0%, CI_95_ [78.8% to 88.4%]). This high proportion remained stable throughout the study period, except for one month (Fig. 1). Of those weighed samples, 196 (95.6%, CI_95_ [91.8% to 97.9%]) were subjectively assessed by blood culture collectors in terms of volume of blood that was drawn. There was a correlation between the time of blood culture sampling and the act of measuring the blood volume collected for culture. The proportion of blood cultures that were not subjected to weighing was significantly greater during the night shifts than the day shifts (24.0% vs. 12.5%; p = 0.039). Similarly, the proportion of blood culture samples that were not weighed was significantly greater during night shifts and/or weekends versus day shifts and/or workdays (22.6% vs. 10.9%; p = 0.022).

Patients’ birthweight and blood sampling site distribution within the study group is presented in Table 1. Of the 205 blood cultures with calculated volume, 24 (11.7%) yielded positive results. There were 15 (7.3% of all weighed samples) true-positive blood cultures and 9 (4.4%) false-positive blood cultures (Table 1).

**Table 1.** Blood culture submissions characteristics

	**n (%)**
Samples weighed	205 (100.0)
Samples with volume estimation	196 (95.6)
Birthweight	205
≤ 1500 g	53 (25.9)
1501–2500 g	90 (43.9)
> 2500 g	62 (30.2)
Material source	205
Cord blood	72 (35.1)
Umbilical catheter	20 (9.8)
Peripheral vessel	112 (54.6)
Not specified	1 (0.5)
Positive blood cultures	24
True-positive blood cultures	15 (62.5)
False-positive blood cultures	9 (37.5)

**Figure S1.**
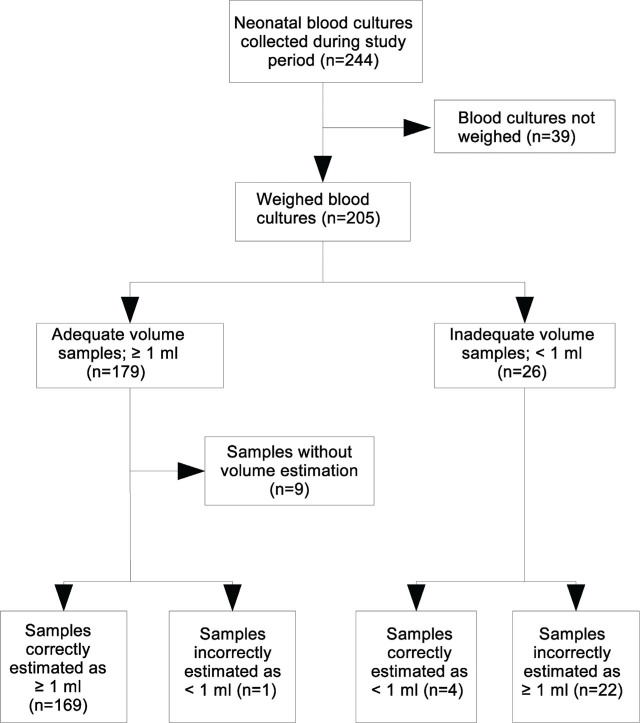
Flow chart of included blood culture submissions throughout the study period.

The percentage of adequate volume samples was 87.3%, CI_95_ [82.0% to 91.5%]. The mean blood volume submitted for culture calculated for the study period was 1.52±0.58 ml. Out of 196 samples visually assessed for collected volume, 5 (2.6%) had an estimation of <1 ml, which was true in 4 cases. The remaining 191 samples (97.4%) were estimated as containing ≥1 ml of blood, which was true in 169 cases (88.5%). Of the 26 inadequate volume samples, 22 were deemed as adequate based on subjective assessment (84.6%, CI_95_ [65.1% to 95.6]). On the other hand, of the 170 adequate samples, there was 1 sample classified subjectively as inadequate (0.6%, CI_95_ [0.01% to 3.2%]). The study flowchart is presented in Supplemental Figure 1. The comparison of blood volume measurement versus blood volume estimation is presented in Table 2.

**Table 2.** Comparison of blood volume measurement versus estimation

		**Blood volume measurement [ml]**		

		**< 1 ml**	**≥ 1 ml**	**Total**
**Blood volume estimation [ml]**	**< 1 ml**	4 (2.0)	1 (0.5)	5 (2.6)
	**≥ 1 ml**	22 (11.2)	169 (86.2)	191 (97.4)
	**Total**	26 (13.3)	170 (86.7)	196 (100.0)

Data presented as n (% of study group).

The blood volumes submitted for culture varied between birthweight categories. Post-hoc analysis confirmed that the volume of samples obtained from patients with a birthweight >2500 g (1.68±0.70 ml) was significantly higher than that from patients weighing ≤1500 g (1.38±0.42 ml) (Supplemental Table 1).

**Figure 2.**
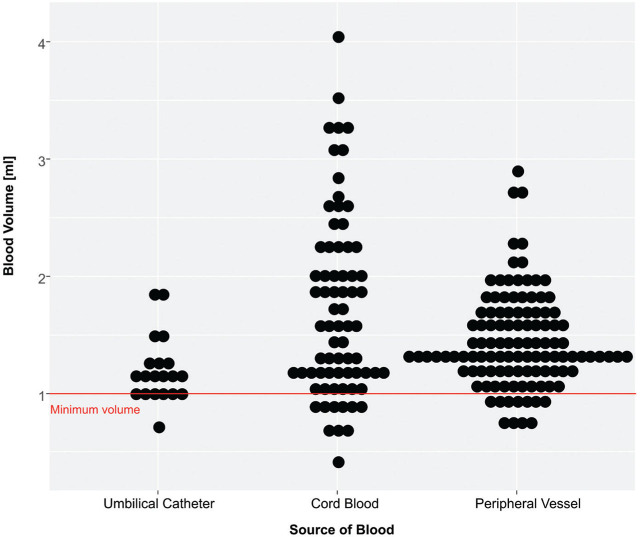
Blood volume summary by source of the material. The horizontal line depicts the minimum blood volume recommended for culture.

The source of blood samples also had a significant impact on the volume of blood that was drawn (p = 0.006). Post-hoc analysis confirmed that the mean volume obtained through umbilical catheters (1.20±0.28 ml) was lower than the volume of cord blood samples (1.72±0.78 ml) or the volume of samples collected from peripheral vessels (1.45±0.40 ml) (Fig. 2, Supplemental Table 2).

Use of the umbilical cord as a source of blood for culture compared to the peripheral vessel did not result in an increased rate of false-positive cultures [4/72 (5.55%) vs. 5/112 (4.46%); p = 0.739] or inadequate submissions [11/72 (15.27%) vs. 11/112 (9.82); p = 0.379]. There were also no significant differences between the proportion of pathogens and contaminants grown with adequate compared to inadequate blood culture volumes (Supplemental Table 3). Blood cultures that yielded true-positive results with inadequate volumes contained > 0.9 ml of blood.

The results of a qualitative questionnaire conducted among medical personnel (14 doctors and 22 nurses) involved in the study are presented in Table 3. Blood culture collection was perceived as an easy procedure by 31% of personnel, while 58% described it as a moderately difficult, and 11% as difficult. A total of 83% of responders answered that verification of blood culture volume by weighing is necessary (for 75%, it was not a substantial impediment; for the remaining 8%, it was a substantial impediment). Two out of 36 responders (5.6%) answered that weighing blood culture samples is unnecessary. There were no significant differences in the assessment of the difficulty of blood culture collection and the necessity of blood sample measurement between doctors and nurses.

**Table 3.** Medical personnel opinion on difficulty of blood culture collection and necessity of blood sample measurement.

	**Total group**	**Doctors**	**Nurses**	**p**
n	36	14	22	
Blood culture collection is:				
Easy	11 (30.6)	6 (42.9)	5 (22.7)	0.469
Moderately difficult	21 (58.3)	7 (50.0)	14 (63.6)	
Difficult	4 (11.1)	1 (7.1)	3 (13.6)	
Don’t know	-	-	-	
Measurement of blood volume collected for culture is:				
Unnecessary	2 (5.6)	-	2 (9.1)	0.244
Necessary, but a substantial impediment	3 (8.3)	1 (7.1)	2 (9.1)	
Necessary and not a substantial impediment	27 (75.0)	13 (92.9)	14 (63.6)	
Don’t know	4 (11.1)	-	4 (18.2)	

Data presented as n (% of study group), groups compared with Fisher exact test.

## Discussion

Bacterial sepsis is still one of the most common clinical problems faced by neonatal care providers. At present, a blood culture remains the gold standard for diagnosing neonatal sepsis, provided that an adequate volume of blood is collected. The phenomenon of “culture-negative” sepsis is likely attributable to falsely negative blood culture results due to insufficient volumes sampled [3]. Harewood et al. demonstrated that despite educational intervention, 39% of 145 cultures from patients less than 1 month of age had a blood volume calculated as 0.0 ml [15]. In such circumstances, negative culture results are obviously worthless, either from a clinical or economical perspective.

Our study showed that point-of-care verification of blood culture volume using a scale was successfully incorporated into routine neonatal practice. The high percentage of blood cultures with calculated volume was observed during the entire study period except for during one month. In November 2020 our unit experienced a shortage of medical staff, including research staff, due to COVID-19, which we believe was responsible for the poor compliance with the intervention. Moreover, the vast majority of medical staff involved in the study reported the intervention as necessary and not as an impediment. All the above findings support the feasibility of conducting a definitive trial regarding the optimization of blood volume submitted for culture in neonates.

We wish to point out that nearly 90% of blood culture submissions in our study contained an adequate volume of blood. We speculate that the specific and immediate feedback to blood culture collectors may support performance in this practical task [16]. The additional benefit offered by our approach was that clinicians taking care of the patients were informed about the potential decreased sensitivity of the results due to suboptimal volume. Conversely, they could rely on negative blood culture results when adequate blood volume was collected. Such information is not available with quality improvement projects based on periodic feedback.

The overestimation of the collected blood volume (understood as calculated volume smaller than estimated) took place in 34% of cases (67/196), whereas the remaining 66% of samples were underestimated (129/196). This was not the case in the group of blood cultures with suboptimal volumes. In this group, more than 80% of blood samples were overly optimistically estimated as adequate submissions. The observed discrepancy could potentially lead to improper clinical decisions.

It is worth noting that drawing blood for culture from isolated cord segments proved to be not inferior to the other two methods in terms of volume adequacy and the rate of contamination. There is a growing body of evidence that cord blood sampling is a painless and blood-saving alternative to initial blood sampling from neonates [17,18,19]. In their randomised study, Balasubramanian et al. demonstrated that this strategy, combined with other anemia prevention efforts, reduced the need for blood transfusions in the neonatal period [18].

We assume that bedside measuring of blood culture volume is a simple and low-budget intervention that could easily be applied in many neonatal units. To achieve better compliance with the intervention, it is important that pre-weighed bottles be immediately available to staff members. Connell et al. observed a significant loss of weight in capped blood culture bottles with time under normal storage conditions [6]. For this reason, we have tested the blood culture bottles we normally used for a possible loss of weight before commencement of this trial. We have noted only negligible loss of weight in the capped glass culture bottles over a period of six months, and a considerable loss of weight in the plastic bottles. We believe that such a test should be performed in any unit willing to incorporate the studied intervention. Moreover, we have decided to use only glass culture bottles in our definitive trial. The surveyed medical staff has tabled no amendments to the intervention. However, our data showed that adherence to the intervention was lower during the night shifts and/or weekends. These findings harmonize with the evidence of the negative impact of the night shifts on overall work performance among healthcare providers [20]. We will conduct monthly feedback and coaching sessions with the staff from each participating unit to overcome this challenge in the definitive trial.

In conclusion, our study showed that point-of-care verification of blood culture volume using a scale is feasible to deliver. The high uptake of the intervention was sustained throughout the study period. Since we have met the pre-established criterion for success, we plan to proceed with the definitive trial.

### Key points

1. Blood cultures remain the gold standard for the diagnosis of neonatal sepsis.
2. A blood volume of at least 1 ml is needed to reliably exclude bacteremia.
3. Volumes of blood submitted for cultures in neonates often do not match the recommended values.
4. It is recommended to introduce methods aimed at verifying the volume of blood that is drawn for culture in neonates.
5. The point-of-care verification of blood culture volume using a scale is feasible to implement into everyday clinical practice.

## Figures and Tables

**Supplemental Table 1.** Volumes submitted for culture according to birthweight category

**Blood volume measurement [ml]**	**Birthweight**			**All birthweight category samples**	**p**

	**≤ 1500 g**	**1501–2500 g**	**> 2500 g**		
Mean ± SD	1.38 ± 0.42^a^	1.49 ± 0.56	1.68 ± 0.70^a^	1.52 ± 0.58	0.004
Range	0.42 – 2.68	0.68 – 3.53	0.69 – 4.05	0.42 – 4.05	

Birthweight groups compared with ANOVA,

a – significant difference in Tukey post-hoc test.

**Supplemental Table 2.** Volumes submitted for culture according to source of the material

**Blood volume measurement [ml]**	**Material source**			**p**

	**Cord blood**	**Umbilical catheter**	**Peripheral vessel**	
Mean ± SD	1.72 ± 0.78^a^	1.20 ± 0.28^a, b^	1.45 ± 0.40^b^	0.006
Range	0.42 – 4.05	0.71 – 1.85	0.71 – 2.90	

Material source groups compared with ANOVA,

a, b – significant difference in Tukey post-hoc test.

**Supplemental Table 3.** Proportion of positivity with adequate and inadequate blood culture volume

**Positive blood cultures**	**Adequate volume cultures ≥ 1 ml**	**Inadequate volume cultures < 1 ml**	**p**
True-positive	13/179 = 7.26%	2/26 = 7.69%	> 0.999
False-positive	8/179 = 4.47%	1/26 = 3.85%	> 0.999

Data presented as n (% of total); groups compared with Fisher exact test.

## References

[1] Puopolo KM, Benitz WE, Zaoutis TE, Committee On F, Newborn, Committee On Infectious D (2018). Management of Neonates Born at >/=35 0/7 Weeks’ Gestation With Suspected or Proven Early-Onset Bacterial Sepsis. Pediatrics.

[2] Shoji K, Tsuboi N, Arakawa R, Ide K, Mikami M, Kato A (2019). Continuous Monitoring and Feedback Optimizes Blood Volume Inoculated Into Culture Bottles in the Pediatric Intensive Care Unit. J Pediatric Infect Dis Soc.

[3] Cantey JB, Baird SD (2017). Ending the Culture of Culture-Negative Sepsis in the Neonatal ICU. Pediatrics.

[4] Schelonka RL, Chai MK, Yoder BA, Hensley D, Brockett RM, Ascher DP (1996). Volume of blood required to detect common neonatal pathogens. J Pediatr.

[5] Buttery JP (2002). Blood cultures in newborns and children: optimising an everyday test. Arch Dis Child Fetal Neonatal Ed.

[6] Connell TG, Rele M, Cowley D, Buttery JP, Curtis N (2007). How reliable is a negative blood culture result? Volume of blood submitted for culture in routine practice in a children's hospital. Pediatrics.

[7] Libertin CR, Sacco KA, Peterson JH (2018). Education and coaching to optimise blood culture volumes: continuous quality improvement in microbiology. BMJ Open Qual.

[8] Ohnishi T, Kamimaki I, Kobayashi R, Nakatogawa K, Amemiya A, Mishima Y (2019). Verification of blood volume for blood culture and detection rate in pediatrics. J Infect Chemother.

[9] Shim H, Kim K, Uh Y, Seo D, Kim H, Yoon Y (2012). The Development and Evaluation of Blood Volume Measuring System for Blood Culture Quality Improvement. Journal of Testing and Evaluation.

[10] Khare R, Kothari T, Castagnaro J, Hemmings B, Tso M, Juretschko S (2020). Active Monitoring and Feedback to Improve Blood Culture Fill Volumes and Positivity Across a Large Integrated Health System. Clin Infect Dis.

[11] Singh MP, Balegar VK, Angiti RR (2020). The practice of blood volume submitted for culture in a neonatal intensive care unit. Arch Dis Child Fetal Neonatal Ed.

[12] Eldridge SM, Chan CL, Campbell MJ, Bond CM, Hopewell S, Thabane L (2016). CONSORT 2010 statement: extension to randomised pilot and feasibility trials. Bmj.

[13] Kenner T (1989). The measurement of blood density and its meaning. Basic Res Cardiol.

[14] Billingham SA, Whitehead AL, Julious SA (2013). An audit of sample sizes for pilot and feasibility trials being undertaken in the United Kingdom registered in the United Kingdom Clinical Research Network database. BMC Med Res Methodol.

[15] Harewood FC, Curtis N, Daley AJ, Bryant PA, Gwee A, Connell TG (2018). Adequate or Inadequate? The Volume of Blood Submitted for Blood Culture at a Tertiary Children's Hospital. Clin Pediatr (Phila).

[16] Archer JC (2010). State of the science in health professional education: effective feedback. Med Educ.

[17] Baer VL, Lambert DK, Carroll PD, Gerday E, Christensen RD (2013). Using umbilical cord blood for the initial blood tests of VLBW neonates results in higher hemoglobin and fewer RBC transfusions. J Perinatol.

[18] Balasubramanian H, Malpani P, Sindhur M, Kabra NS, Ahmed J, Srinivasan L (2019). Effect of Umbilical Cord Blood Sampling versus Admission Blood Sampling on Requirement of Blood Transfusion in Extremely Preterm Infants: A Randomized Controlled Trial. J Pediatr.

[19] Greer R, Safarulla A, Koeppel R, Aslam M, Bany-Mohammed FM (2019). Can Fetal Umbilical Venous Blood Be a Reliable Source for Admission Complete Blood Count and Culture in NICU Patients?. Neonatology.

[20] Caruso CC (2014). Negative impacts of shiftwork and long work hours. Rehabil Nurs.

